# Supplementary material for: Lipoarabinomannan mediates localized cell wall integrity during division in mycobacteria
Source: Nat Commun. 2024 Mar 11;15:2191. doi: 10.1038/s41467-024-46565-5 (PMC10928101; doi:10.1038/s41467-024-46565-5)
Supplement: Supplementary file 1 — Supplementary Information [file 41467_2024_46565_MOESM1_ESM.pdf]

Supplemental Information for

Lipoarabinomannan mediates localized cell wall integrity during division in mycobacteria

Ian L. Sparks[1], Takehiro Kado[1], Malavika Prithviraj[1], Japinder Nijjer[2,3], Jing Yan[2,3], and Yasu S. Morita[1]*

[1] Department of Microbiology, University of Massachusetts, Amherst, MA, USA. [2] Department of Molecular, Cellular and Developmental Biology, Yale University, New Haven, CT, USA. [3] Quantitative Biology Institute, Yale University, New Haven, CT, USA.

* ymorita@umass.edu

This PDF file includes:

Figures S1 to S4
Table S1 to S3

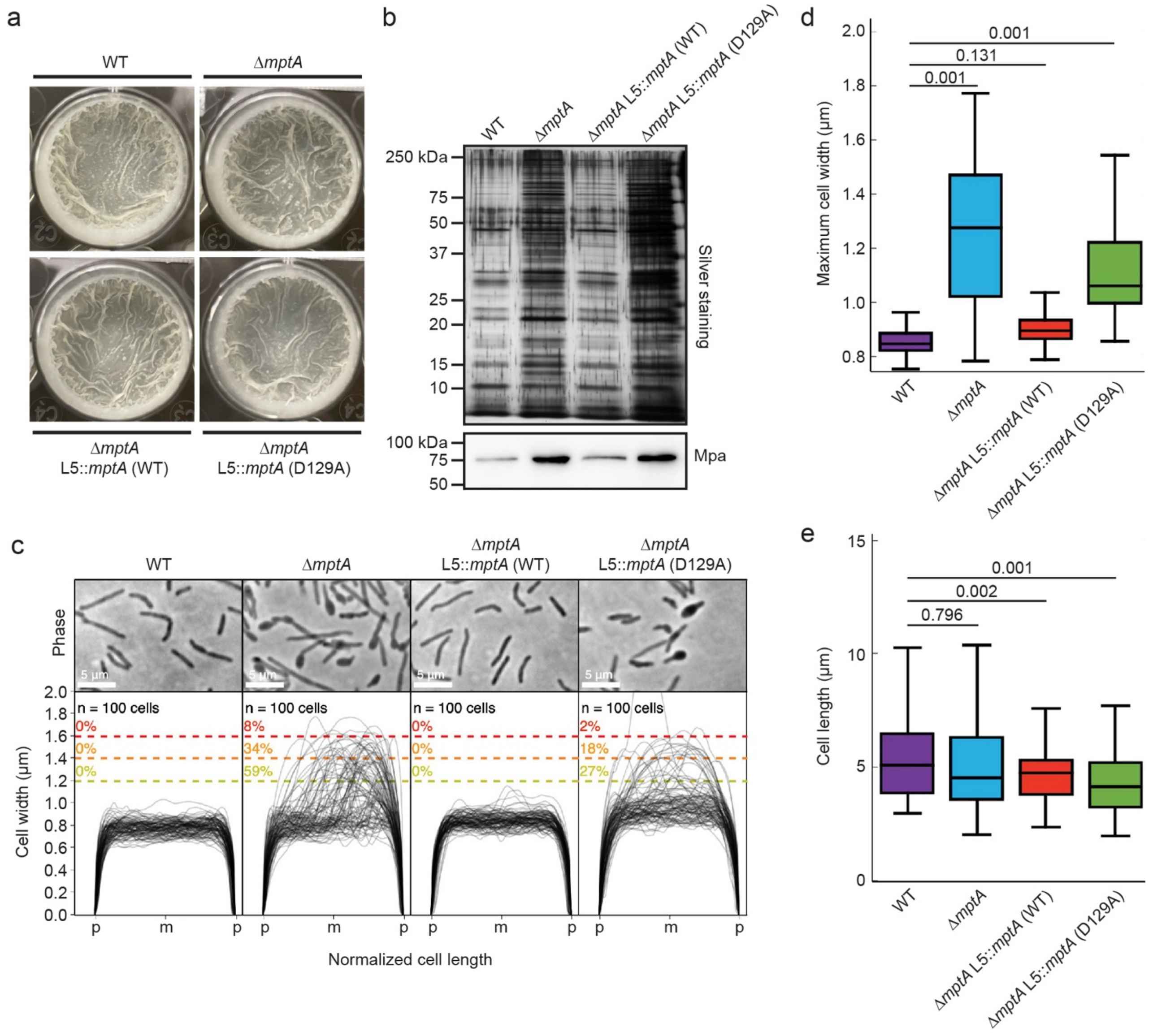

Figure S1. MptA-deficient cells lyse in pellicle growth. a) Top-down view of WT, Δ*mptA,* Δ*mptA* L5::*mptA* (WT), and Δ*mptA* L5::*mptA* (D129A) pellicle biofilms after 5-day growth in M63 at 37°C. **b)** SDS-PAGE of culture filtrates from WT, Δ*mptA,* Δ*mptA* L5::*mptA* (WT), and Δ*mptA* L5::*mptA* (D129A) pellicles visualized by silver staining (top) and anti-Mpa (cytoplasmic marker protein) immunoblotting (bottom). **c)** Phase contrast micrographs and cell width profiles of WT, Δ*mptA,* Δ*mptA* L5::*mptA* (WT), and Δ*mptA* L5::*mptA* (D129A) pellicles grown in M63 medium. Each cell's length was normalized to the same length with "p" and "m" indicating cell poles and mid-cell, respectively. The percentage values above the dotted colored lines indicate the portion of cells exhibiting maximum cell widths greater than or equal to the corresponding cell width threshold. **d and e)** Boxplots comparing the distribution of maximum cell widths (d) and cell lengths (e) of WT, Δ*mptA,* Δ*mptA* L5::*mptA* (WT), and Δ*mptA* L5::*mptA* (D129A) cells grown as pellicles in M63 medium. A box spanning the interquartile range (IQR) is drawn from the first quartile to the third quartile with a horizontal line indicating the median. The whiskers extend from the box to the farthest data point within 1.5xIQR from the box. Statistical significance was determined by ANOVA and Tukey post-hoc test. n = 100 cells for each strain in panel d and n = 100, 112, 157, 135 cells for WT, Δ*mptA*, Δ*mptA* L5::*mptA* (WT), and Δ*mptA* L5::*mptA* (D129A) respectively in panel e. Source data for panels b-e are provided in the Source Data file. Macro and Microscopy image data for panels a, c, d, and e are available at https://github.com/IanLairdSparks/Sparks_2023.

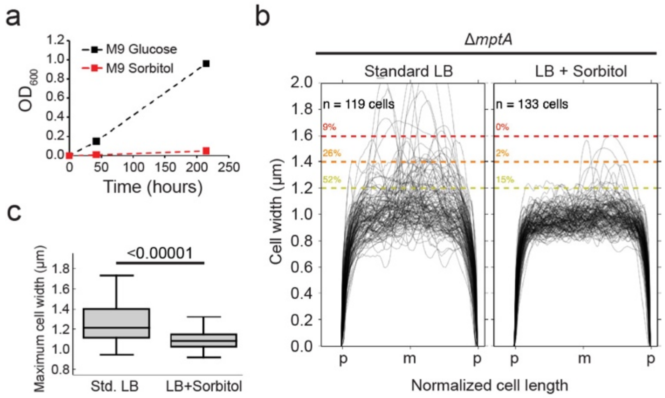

Figure S2. Defective cell morphology of ∆*mptA* grown planktonically in LB broth was alleviated by sorbitol. a) Growth curve, measured by optical density at 600 nm, of WT *M. smegmatis* in standard M9 minimum medium (containing 22 mM glucose as a sole carbon source; black) or modified M9 (containing 22 mM sorbitol as a sole carbon source; red), showing that *M. smegmatis* cannot grow on sorbitol as a sole carbon source. **b)** Cell width profiles of ∆*mptA* cells grown planktonically in LB and osmo-protective LB (supplemented with 500 mM sorbitol). Normalized cell width profile was graphed as described in Fig. S1c. **c)** Boxplot comparing the distribution of maximum cell widths between ∆*mptA* cells planktonically grown in standard vs. osmo-protective LB broth. Boxplots are drawn as described in Fig. S1d and e. Statistical significance was determined using the student's T-test. $P = 3.22 \times 10^{-12}$. n = 119 and 133 cells for ∆*mptA* in standard LB and LB + sorbitol respectively. Source data for each sub panel are provided in the Source Data file. Microscopy image data for panels b and c are available at https://github.com/IanLairdSparks/Sparks_2023.

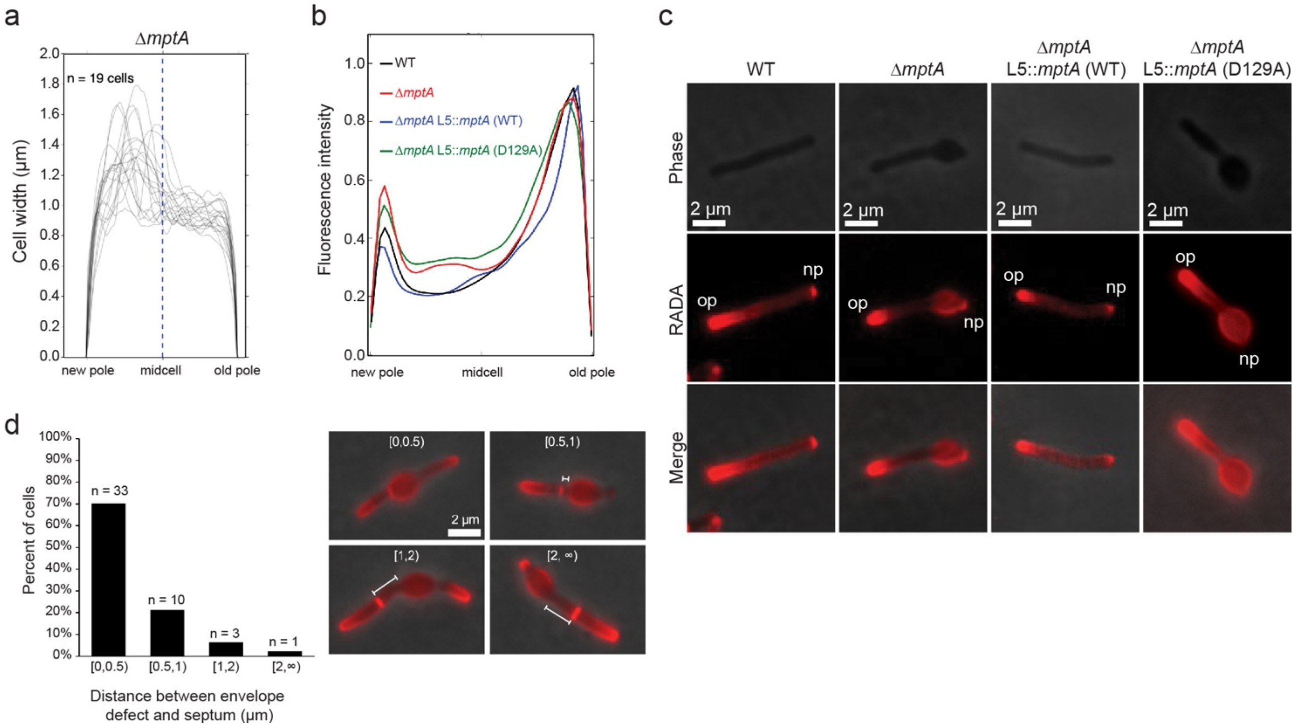

Figure S3. Cell envelope defects associate with the new pole and septa. a) New-pole-aligned cell width profiles of deformed, non-septated Δ*mptA* cells grown planktonically in LB medium. Cells were labelled with RADA and aligned from the new pole (dim) to the old pole (bright) based on RADA labelling. **b)** New-pole-aligned RADA labelling profile of WT, Δ*mptA,* Δ*mptA* L5::*mptA* (WT), and Δ*mptA* L5::*mptA* (D129A) cells grown planktonically in LB medium (n = 66, n = 53, n = 64, n = 35 respectively). Only non-septated cells were selected for the analysis. **c)** Example phase-contrast and fluorescence microscopy images of RADA-labelled WT, Δ*mptA,* Δ*mptA* L5::*mptA* (WT), and Δ*mptA* L5::*mptA* (D129A) cells. np, the new pole; op, the old pole. **d)** Quantification of the distance between RADA-labeled cell envelope deformations and the septum in 47 septated Δ*mptA* cells. The cell length of 47 septated cells ranged from 3.2 to 9.2 μm. Distances were binned into four categories: less than 0.5 μm (denoted as [0,0.5)), greater than or equal to 0.5 μm and less than 1 μm (denoted as [0.5,1)), greater than or equal to 1 μm and less than 2 μm (denoted as [1,2)), or greater than or equal to 2 μm (denoted as [2,∞)). The micrographs depict example merged phase-contrast/fluorescence images of RADA-labeled Δ*mptA* cells for each bin. White line indicates the measured distance for each cell. Source data for panels a, b, and d are provided in the Source Data file. Microscopy image data for each sub panel are available at https://github.com/IanLairdSparks/Sparks_2023.

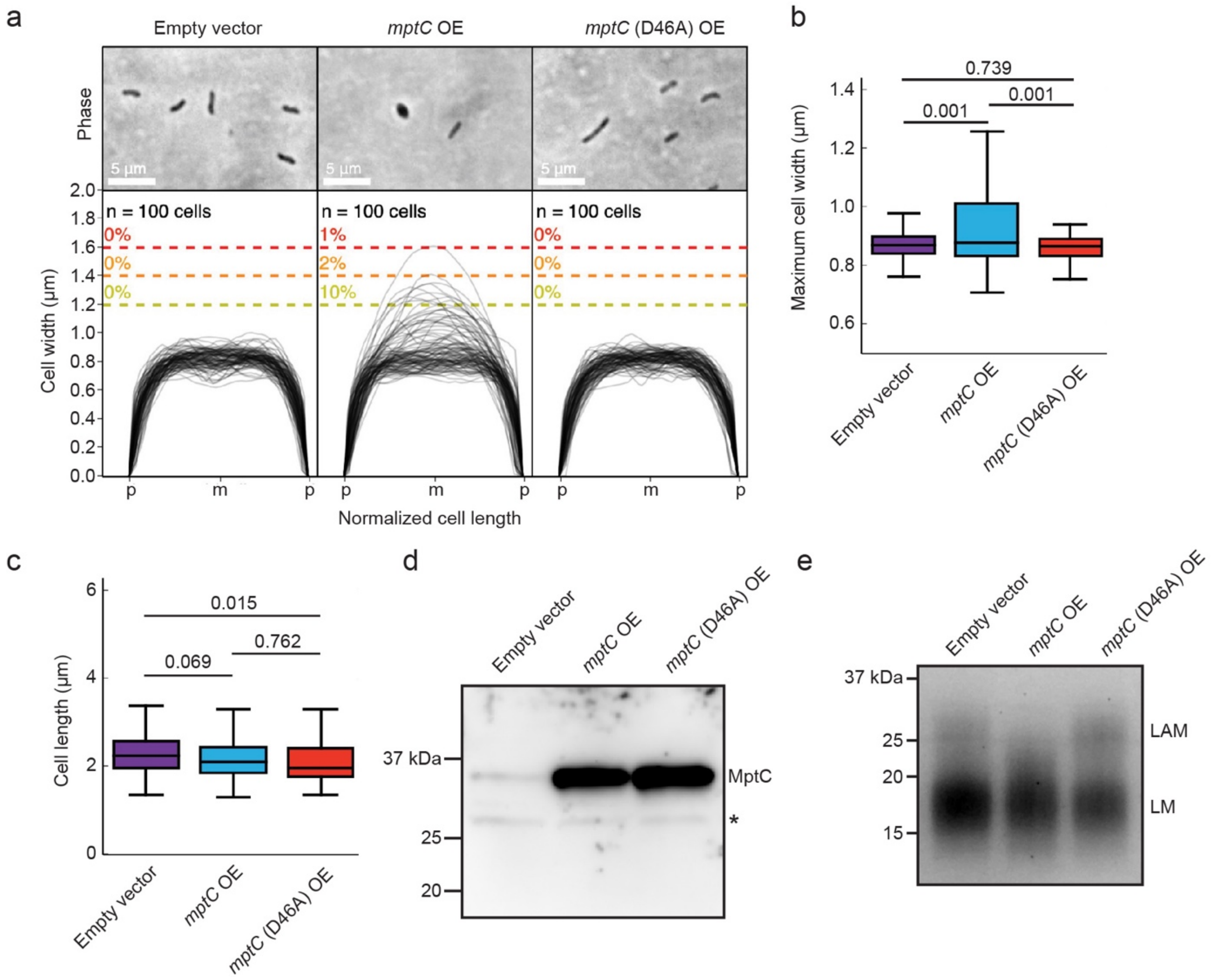

Figure S4. *mptC* overexpression induces cell shape defects in *M. tuberculosis*. a) Phase contrast micrographs and cell width profiles of *M. tuberculosis* transformed with empty vector control, *mptC* overexpression vector (*mptC* OE), and catalytically inactive *mptC* overexpression vector (*mptC* (D46A) OE) grown in Middlebrook 7H9. Cell width profiles were graphed as described in Fig. S1c. **b and c)** Boxplots comparing the distribution of maximum cell widths (b) and cell lengths (c) of *mptC* OE, *mptC* (D46A) OE, and empty vector control strains grown in Middlebrook 7H9. Boxplots are drawn as described in Fig. S1d and e. Statistical significance was determined by ANOVA and Tukey post-hoc test. n = 100 cells for each strain in panel b and n = 218, 138, 109 cells for empty vector control, *mptC* OE, and *mptC* (D46A) OE respectively in panel c. **d)** Anti-MptC immunoblot of cell lysates prepared from *mptC* OE, *mptC* (D46A) OE, and empty vector control strains grown in Middlebrook 7H9. The asterisk indicates a nonspecific band. **e)** SDS-PAGE of lipoglycans extracted from *mptC* OE, *mptC* (D46A) OE, and empty vector control *M. tuberculosis* strains, visualized by glycan staining. Source data for each sub panel are provided in the Source Data file. Microscopy image data for panels a-c are available at https://github.com/IanLairdSparks/Sparks_2023.

Antibiotic (+sulbactam)	MIC (µg/ml) WT	MIC (µg/ml) Δ*mptA*	Fold change in sensitivity (Δ*mptA* / WT)
Isoniazid	25.0	20.8	1.2
Vancomycin	0.8	0.4	2.0
Moenomycin	0.8	0.2	4.0
Ampicillin	8.3	0.2	42.7
Meropenem	6.3	0.8	8.0
D-cycloserine	83.3	50.0	1.7
Kanamycin	2.1	0.8	2.7

Table S1. Δ*mptA* is more sensitive to β-lactam antibiotics. Cells were grown in Middlebrook 7H9. MIC, minimum inhibitory concentration. A non-growth-inhibitory dose of 50 µg/mL sulbactam was added to inhibit β-lactamases. Known primary drug targets are: isoniazid, the outer membrane biosynthesis; vancomycin and moenomycin, cell wall transglycosylation; ampicillin, cell wall transpeptidation (primarily D-D crosslinks); meropenem, cell wall transpeptidation (primarily L-D crosslinks); D-cycloserine, cytoplasmic cell wall precursor synthesis; and kanamycin, ribosome. Source data are provided in the Source Data file.

Antibiotic (+sulbactam)	MIC (µg/ml) In LB		Fold change in sensitivity (Δ*mptA* / WT)	MIC (µg/ml) in LB + Sorbitol		Fold change in sensitivity (Δ*mptA* / WT)
	WT	Δ*mptA*		WT	Δ*mptA*	
Ampicillin	200	2.3	85.3	200	12.5	16
Meropenem	50	0.39	128.2	200	6.25	32
Kanamycin	3.1	1.6	2	3.1	2.3	1.3

Table S2. Antibiotic sensitivity of Δ*mptA* is pronounced in LB and the enhanced antibiotic susceptibility is alleviated by sorbitol supplementation. MIC, minimum inhibitory concentration. A non-growth-inhibitory dose of 50 µg/mL sulbactam was added to inhibit β-lactamases. Source data are provided in the Source Data file.

Antibiotic (+sulbactam)	MIC (µg/ml) In LB		
	Δ*mptA*	Δ*mptA* *L5::mptA* (WT)	Δ*mptA* *L5::mptA (D129A)*
Ampicillin	1.56	18.75	1.56
Meropenem	0.39	3.13	1.56

Table S3. Antibiotic sensitivity of Δ*mptA* is restored by WT *mptA* complementation. MIC, minimum inhibitory concentration. A non-growth-inhibitory dose of 50 µg/mL sulbactam was added to inhibit β-lactamases. Source data are provided in the Source Data file.
