# Supplementary material for: Lipoarabinomannan mediates localized cell wall integrity during division in mycobacteria
Source: Nat Commun. 2024 Mar 11;15:2191. doi: 10.1038/s41467-024-46565-5 (PMC10928101; doi:10.1038/s41467-024-46565-5)
Supplement: Supplementary file 3 — Description of Additional Supplementary Files [file 41467_2024_46565_MOESM3_ESM.pdf]

Description of Additional Supplementary Files:

Supplementary Data 1: Statistical parameters for one-way ANOVA and Turkey HSD post-hoc test. For comparisons of more than two means, statistical significance was determined by ANOVA and Tukey HSD post-hoc test, calculated by the “One-way ANOVA (ANalysis Of VAriance) with post-hoc Tukey HSD (Honestly Significant Difference) Test Calculator for comparing multiple treatments” tool (astatsa.com, 2016 Navendu Vasavada). Test parameters for each experiment are arranged in a large table, with the experiment identifier in the left-most cell (example: “Fig. 1e 30°C 7H10”). ANOVA parameters are shown directly right of the experiment identifier, and include the names of the conditions tested (example: “ΔmptA”), the placeholder name of each condition for use in the Turkey post-hoc test (example: “B”), the sample size for each test condition (example: “n = 22”), The critical F value for a significance threshold (alpha) = 0.05, the F statistic, and the P value. Tukey post-hoc test parameters are shown directly right of the ANOVA parameters and include the critical Q value for a significance threshold (alpha) = 0.05, the comparisons tested (example: “A vs B”, using placeholder names of test conditions for simplicity), the Q statistic for each comparison, and the P value for each comparison.
