# Supplementary material for: Lipoarabinomannan mediates localized cell wall integrity during division in mycobacteria
Source: Nat Commun. 2024 Mar 11;15:2191. doi: 10.1038/s41467-024-46565-5 (PMC10928101; doi:10.1038/s41467-024-46565-5)
Supplement: Supplementary file 5 — Reporting Summary [file 41467_2024_46565_MOESM5_ESM.pdf]

nature portfolio

Corresponding author(s): Yasu S. Morita

Last updated by author(s): 2/25/2024

Reporting Summary

Nature Portfolio wishes to improve the reproducibility of the work that we publish. This form provides structure for consistency and transparency in reporting. For further information on Nature Portfolio policies, see our Editorial Policies and the Editorial Policy Checklist.

Statistics

For all statistical analyses, confirm that the following items are present in the figure legend, table legend, main text, or Methods section.

n/a	Confirmed	
☐	☒	The exact sample size (*n*) for each experimental group/condition, given as a discrete number and unit of measurement
☐	☒	A statement on whether measurements were taken from distinct samples or whether the same sample was measured repeatedly
☐	☒	The statistical test(s) used AND whether they are one- or two-sided<br>*Only common tests should be described solely by name; describe more complex techniques in the Methods section.*
☐	☒	A description of all covariates tested
☐	☒	A description of any assumptions or corrections, such as tests of normality and adjustment for multiple comparisons
☐	☒	A full description of the statistical parameters including central tendency (e.g. means) or other basic estimates (e.g. regression coefficient) AND variation (e.g. standard deviation) or associated estimates of uncertainty (e.g. confidence intervals)
☐	☒	For null hypothesis testing, the test statistic (e.g. *F*, *t*, *r*) with confidence intervals, effect sizes, degrees of freedom and *P* value noted<br>*Give P values as exact values whenever suitable.*
☒	☐	For Bayesian analysis, information on the choice of priors and Markov chain Monte Carlo settings
☒	☐	For hierarchical and complex designs, identification of the appropriate level for tests and full reporting of outcomes
☒	☐	Estimates of effect sizes (e.g. Cohen's *d*, Pearson's *r*), indicating how they were calculated

Our web collection on statistics for biologists contains articles on many of the points above.

Software and code

Policy information about availability of computer code

Data collection	Oufti (version 1), Matlab (version R2022b)
Data analysis	R (version 4.0.3), ggplot2 (version 3.3.3), Python (version 2.7.16), Python package NumPy (version 1.8.0rc1), matplotlib (version 1.3.1), custom python script (https://github.com/IanLairdSparks/Sparks_2023), Microsoft Excel (version 16.77.1).

For manuscripts utilizing custom algorithms or software that are central to the research but not yet described in published literature, software must be made available to editors and reviewers. We strongly encourage code deposition in a community repository (e.g. GitHub). See the Nature Portfolio guidelines for submitting code & software for further information.

Data

Policy information about availability of data

All manuscripts must include a data availability statement. This statement should provide the following information, where applicable:

- Accession codes, unique identifiers, or web links for publicly available datasets
- A description of any restrictions on data availability
- For clinical datasets or third party data, please ensure that the statement adheres to our policy

Source data are provided with this paper. The data shown in tables, dot plots, scatter plots, and line plots in main and supplementary figures are provided in the Source Data file. All original gel/blot images are also available in the Source Data file. Raw image data for Fig. 1f can be accessed through Dryad (https://

doi.org/10.5061/dryad.1vhhmgr1w). All other image data used to generate data in this study are available from GitHub under https://github.com/IanLairdSparks/Sparks_2023.

Research involving human participants, their data, or biological material

Policy information about studies with human participants or human data. See also policy information about sex, gender (identity/presentation), and sexual orientation and race, ethnicity and racism.

Reporting on sex and gender	N/A
Reporting on race, ethnicity, or other socially relevant groupings	N/A
Population characteristics	N/A
Recruitment	N/A
Ethics oversight	N/A

Note that full information on the approval of the study protocol must also be provided in the manuscript.

Field-specific reporting

Please select the one below that is the best fit for your research. If you are not sure, read the appropriate sections before making your selection.

[x] Life sciences [ ] Behavioural & social sciences [ ] Ecological, evolutionary & environmental sciences

For a reference copy of the document with all sections, see nature.com/documents/nr-reporting-summary-flat.pdf

Life sciences study design

All studies must disclose on these points even when the disclosure is negative.

Sample size	Data from pilot experiments were used to determine that the observed effect sizes of our observed phenotypes were large (Cohen's d > 0.8). Power analysis was then applied to determine the appropriate sample size for each experiment while maintaining a minimum statistical power level of 0.80 (80% chance of correctly rejecting the null hypothesis; the standard power level used for power analysis) and a significance threshold (alpha) of 0.05 while assuming an effect size of 0.8 (the widely accepted value for a large effect size, conservatively less than our own measured effect sizes). Actual sample sizes in our experiments are equal to or greater than the sample sizes suggested by power analysis, ensuring strong statistical power throughout our study.
Data exclusions	Overlapping cells were excluded from microscopy analysis.
Replication	All experiments were repeated at least twice, with representative analysis shown. All attempts at replication were successful.
Randomization	For conditions in which more than 100 cells were analyzed, a randomized subset of 100 cells were used.
Blinding	All experiments are done individually by a single researcher, and blinding was impractical for the type and scale of the experiments conducted in our laboratories.

Reporting for specific materials, systems and methods

We require information from authors about some types of materials, experimental systems and methods used in many studies. Here, indicate whether each material, system or method listed is relevant to your study. If you are not sure if a list item applies to your research, read the appropriate section before selecting a response.

Materials & experimental systems

n/a	Involved in the study	
[ ]	[x]	Antibodies
[x]	[ ]	Eukaryotic cell lines
[x]	[ ]	Palaeontology and archaeology
[x]	[ ]	Animals and other organisms
[x]	[ ]	Clinical data
[x]	[ ]	Dual use research of concern
[x]	[ ]	Plants

Methods

n/a	Involved in the study	
[x]	[ ]	ChIP-seq
[x]	[ ]	Flow cytometry
[x]	[ ]	MRI-based neuroimaging

Antibodies

Antibodies used	Anti-M. smegmatis MptA (from rabbit) (Custom-made, Medical and Biological Laboratories, Tokyo, Japan) (PMID: 20215111)<br>Anti-M. tuberculosis MptA (from rabbit) (Custom-made, Medical and Biological Laboratories, Tokyo, Japan) (this study)<br>Anti-M. tuberculosis MptC (from rabbit) (Custom-made, Medical and Biological Laboratories, Tokyo, Japan) (PMID: 23422411)<br>Anti-M. smegmatis Mpa (from rabbit) (Gift from Dr. Heran Darwin, New York University) (PMID: 15659170)<br>Anti-rabbit IgG Horseradish Peroxidase linked F(ab')2 fragment (from donkey)(Cytiva, Cat# NA9340V, Lot# 6969617)
Validation	Anti-M. smegmatis MptA: validated by 1) binding to an affinity column made with peptides that are used to raise the antibody and 2) expected quantitative changes of a band detected near the expected molecular weight (54.3 kDa) in response to gene overexpression in M. smegmatis (PMID: 20215111) and gene knockdown in M. smegmatis (PMID: 23422411).<br><br>Anti-M. tuberculosis MptA: validated by 1) binding to an affinity column made with peptides that are used to raise the antibody and 2) the expected quantitative changes of a band detected near the expected molecular weight (55.3 kDa) in response to gene knockdown (this study).<br><br>Anti-M. tuberculosis MptC: validated by 1) binding to an affinity column made with peptides that are used to raise the antibody and 2) the expected quantitative changes of a band detected near the expected molecular weight (47.1 kDa) in response to gene knockout in M. tuberculosis (PMID: 23422411) and gene overexpression in M. tuberculosis (PMID: 23422411).<br><br>Anti-M. smegmatis Mpa: We used this antibody (gift from Dr. Heran Darwin, New York University) as a cytoplasmic marker. We have validated the protein's cytoplasmic localization in M. smegmatis by subcellular fractionation in our previous study (PMID: 28855252).
